# Supplementary material for: Randomizing bipartite networks: the case of the World Trade Web
Source: Sci Rep. 2015 Jun 1;5:10595. doi: 10.1038/srep10595 (PMC4450581; doi:10.1038/srep10595)
Supplement: Supplementary Information [file srep10595-s1.pdf]

Randomizing bipartite networks: the case of the World Trade Web Supplementary Information

Fabio Saracco,[1] Riccardo Di Clemente,[1] Andrea Gabrielli,[1,2,3] and Tiziano Squartini[1]

[1] *Istituto dei Sistemi Complessi (ISC) - CNR, UoS Sapienza, Dipartimento di Fisica, Universitá "Sapienza" di Roma, P.le A. Moro 5, 00185 Roma (Italy)*

[2] *IMT Institute for Advanced Studies, P.zza S. Ponziano 6, 55100 Lucca (Italy)*

[3] *INFN - Unitá Roma1, Dipartimento di Fisica, Universitá "Sapienza" di Roma, P.le A. Moro 5, 00185 Roma (Italy)*

(Dated: March 17, 2015)

Supplementary Information for the paper "Randomizing bipartite networks: the case of the World Trade Web".

PACS numbers: 89.75.Da; 02.50.Le; 89.65.Ef

I. THE RANDOM GRAPH MODEL

In the main text we have explicitly shown only the first and last passages of the calculations for the bipartite Configuration Model. The full passages are reported below:

$$P(\mathbf{M}|\vec{\theta}) = \frac{e^{-\sum_{c,p}(\alpha_c+\beta_p)m_{cp}(\mathbf{M})}}{\sum_{\mathbf{M}} e^{-\sum_{c,p}(\alpha_c+\beta_p)m_{cp}(\mathbf{M})}} = \frac{\prod_{c,p} e^{-(\alpha_c+\beta_p)m_{cp}(\mathbf{M})}}{\sum_{\mathbf{M}}\prod_{c,p} e^{-(\alpha_c+\beta_p)m_{cp}(\mathbf{M})}} = \frac{\prod_{c,p}(x_c y_p)^{m_{cp}(\mathbf{M})}}{\prod_{c,p}(1+x_c y_p)}$$
$$= \prod_{c,p} p_{cp}^{m_{cp}}(1-p_{cp})^{1-m_{cp}} \qquad \text{(I.1)}$$

(with $e^{-\alpha_c} = x_c$, $e^{-\beta_p} = y_p$ and $p_{cp} = \frac{x_c y_p}{1+x_c y_p}$). The calculations for the BiRG proceed along the same lines of those for the BiCM:

$$P(\mathbf{M}|\theta) = \frac{e^{-\theta\sum_{c,p} m_{cp}(\mathbf{M})}}{\sum_{\mathbf{M}} e^{-\theta\sum_{c,p} m_{cp}(\mathbf{M})}} = \frac{\prod_{c,p} e^{-\theta m_{cp}(\mathbf{M})}}{\sum_{\mathbf{M}}\prod_{c,p} e^{-\theta m_{cp}(\mathbf{M})}} = \frac{x^{L(\mathbf{M})}}{\prod_{c,p}(1+x)} \qquad \text{(I.2)}$$

(with $e^{-\theta} = x$). Some more algebra leads to

$$P(\mathbf{M}|x) = \left(\frac{x}{1+x}\right)^{L(\mathbf{M})}\left(\frac{1}{1+x}\right)^{C\cdot P-L(\mathbf{M})} = p^{L(\mathbf{M})}(1-p)^{C\cdot P-L(\mathbf{M})} \qquad \text{(I.3)}$$

with $x/(1+x) = p$. Maximizing the network log-likelihood function leads to the result $p = c(\mathbf{M}) = L(\mathbf{M})/C\cdot P$.

II. TOPOLOGICAL MEASURES FOR BINARY, UNDIRECTED, BIPARTITE NETWORKS

A. Complexity and fitness

In order to infer the productive properties of the different countries from the incidence matrix $\mathbf{M}$, in the context of Economic Complexity [7–9], the fitness and complexity algorithm has been proposed in [8]; roughly speaking, it is a generalization of the Google PageRank to bipartite networks. The algorithm assigns high fitness to the countries exporting the most exclusive (i.e. with higher complexity) products. In particular, the fitness F_c for country c and the complexity Q_p for product p are defined, at the n-th iteration of the algorithm, as

$$\begin{cases} \tilde{F}_c^{(n)} = \sum_{p=1}^{P} m_{cp} Q_p^{(n-1)} \\ \tilde{Q}_p^{(n)} = \dfrac{1}{\sum_{c=1}^{C} m_{cp}\frac{1}{F_c^{(n-1)}}} \end{cases} \rightarrow \begin{cases} F_c^{(n)} = \dfrac{\tilde{F}_c^{(n)}}{\langle \tilde{F}_c^{(n)}\rangle} \\ Q_p^{(n)} = \dfrac{\tilde{Q}_p^{(n)}}{\langle \tilde{Q}_p^{(n)}\rangle} \end{cases}, \qquad \text{(II.1)}$$

where the symbols $\langle\dots\rangle$ indicate the averages taken over the sets $\{\tilde{F}_c^{(n)}\}_{c=1}^{C}$ and $\{\tilde{Q}_p^{(n)}\}_{p=1}^{P}$. The initial conditions can be chosen to be $F_c^0 = Q_p^0 = 1$, $\forall c$, $\forall p$. Further details on the convergence of the algorithm in (II.1) can be found in [45]. The non-linear behaviour of fitness and complexity can be highlighted by respectively comparing the value of the diversification d_c (ubiquity u_p) with the ranking obtained through the fitness F_c (complexity Q_p) values, as shown in fig. 3 of the Main Text.

B. Nestedness

Several different definitions of nestedness can be encountered in literature [41–44]. In the present article we use the definition called NODF (an acronym for *Nestedness metric based on Overlap and Decreasing Fill*) and proposed in [41]. Let us define

$$S_{c\,c'} = \begin{cases} d_c \neq d_{c'} & \dfrac{\sum_p m_{c\,p}\, m_{c'\,p}}{\min\{d_c, d_{c'}\}} \\ \text{otherwise} & 0 \end{cases}, \quad T_{p\,p'} = \begin{cases} u_p \neq u_{p'} & \dfrac{\sum_c m_{c\,p}\, m_{c\,p'}}{\min\{u_p, u_{p'}\}} \\ \text{otherwise} & 0 \end{cases}. \tag{II.2}$$

Notice that $S_{c\,c'}$ ($T_{p\,p'}$) are solely determined by those pairs of countries (products) for which the number of ones in rows c and c' (in columns p and p') are different. The measure of nestedness called NODF is then defined as

$$\text{NODF} = 2\frac{\sum_{c<c'} S_{c\,c'} + \sum_{p<p'} T_{p\,p'}}{C(C-1) + P(P-1)}. \tag{II.3}$$

The definition (II.3) results from summing the contribution coming from rows and from columns, being normalized to the total number of couples of rows and columns. In order to isolate the single contributions coming from rows and columns, it is possible to defined the countries-specific and the products-specific NODF, respectively as

$$\text{NODF}_c = 2\frac{\sum_{c<c'} S_{c\,c'}}{C(C-1)}, \;\; \text{NODF}_p = 2\frac{\sum_{p<p'} T_{p\,p'}}{P(P-1)}. \tag{II.4}$$

III. EXPECTED TOPOLOGICAL MEASURES FOR BINARY, UNDIRECTED, BIPARTITE NETWORKS

A. Assortativity

The expected value of the assortativity coefficients is easily calculable, after noticing that $\langle d_c \rangle = d_c$ and $\langle u_p \rangle = u_p$ by construction, that $m_{cp}^2 = m_{cp}$, being m_{cp} a binary variable, and resting upon the approximation $\langle \frac{n}{d} \rangle \simeq \frac{\langle n \rangle}{\langle d \rangle}$:

$$\langle u_c^{nn} \rangle = \frac{\sum_{p=1}^{P} p_{cp}(u_p - p_{cp} + 1)}{d_c}, \;\; \langle d_p^{nn} \rangle = \frac{\sum_{c=1}^{C} p_{cp}(d_c - p_{cp} + 1)}{u_p}. \tag{III.1}$$

Assortativity standard deviation can be calculated by applying the so-called *delta method*, whose generic formula reads

$$\sigma_X \simeq \sqrt{\sum_{c=1}^{C}\sum_{p=1}^{P} \left(\frac{\partial X(\mathbf{M})}{\partial m_{cp}} \right)^2_{m_{cp}=p_{cp}} \sigma^2_{m_{cp}}}, \tag{III.2}$$

providing a method to calculate the standard deviation of any function of interest, $X(\mathbf{M})$, in terms of the independent random variables (in our case the entries m_{cp} of the incidence matrix).

B. Motifs

In addition to the Vn and Λn family we can define more complex motifs, enlarging the number of nodes of the two layers to be considered. For example, X-*motifs* can be defined, i.e. combinations of two V-motifs subtending the same pairs of countries and products (see fig. 2 in the Main Text):

$$N_X(\mathbf{M}) = \sum_{c<c'} \sum_{p<p'} m_{cp} m_{cp'} m_{c'p} m_{c'p'} = \sum_{c<c'} \binom{\mathcal{C}_{cc'}}{2} = \sum_{p<p'} \binom{\mathcal{P}_{pp'}}{2} \tag{III.3}$$

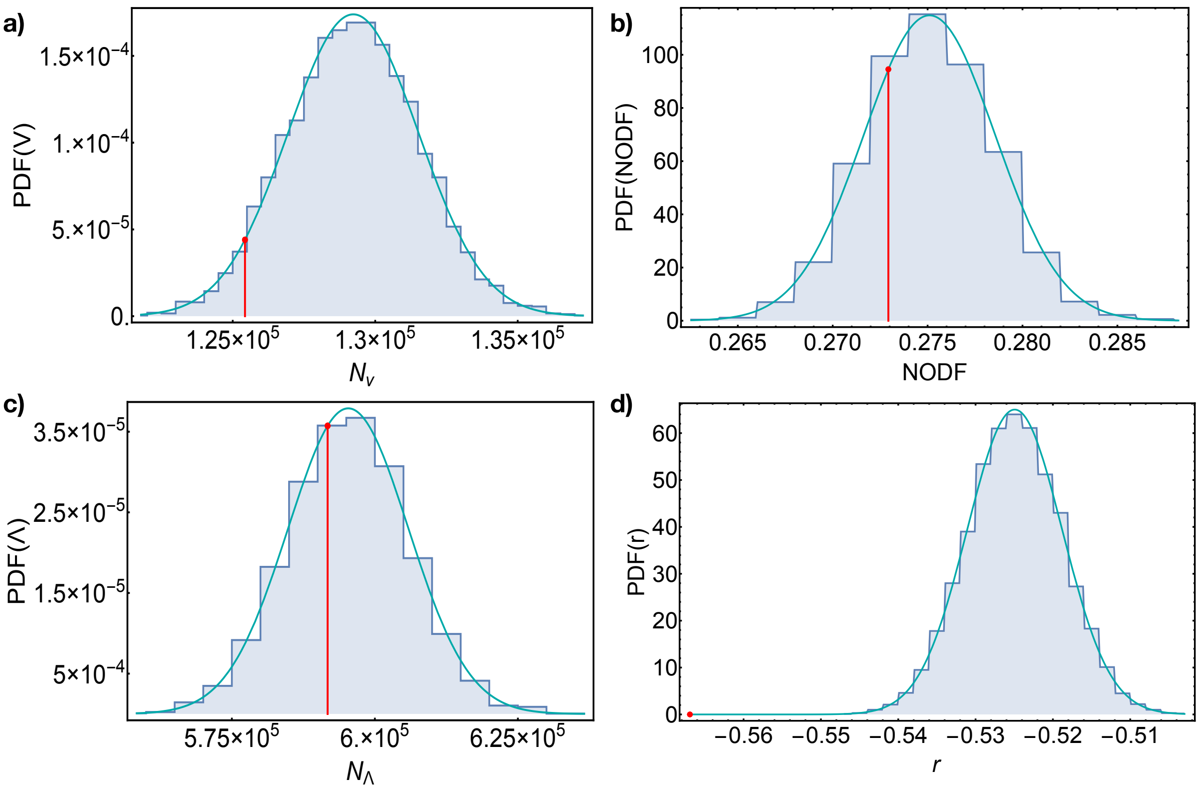

FIG. 1. Ensemble distribution of N_V and N_Λ abundance (panels a and c), assortativity coefficient r and NODF (panels b and d) in the year 2000 (fits are obtained by superimposing normal distributions with the sample average and variance; red points represent the observed motifs abundance).

(the notation $\sum_{c<c'}$ being equivalent to $\sum_{c=1}^{C}\sum_{c'=c+1}^{C}$ and similarly for products). As evident from the definition, X-motifs measure the co-occurrence of two countries as producers of the same pair of products and, viceversa, the co-occurrence of two products in the baskets of the same two countries. Thus, competitiveness on different segments of the market can now be measured, refining the information provided by V-motifs.

Allowing for an even higher number of nodes to interact, M-*motifs* and W-*motifs* can be defined (see fig. 2 in the Main Text) as

$$N_M(\mathbf{M}) = \sum_{c<c'}\sum_{p<p'<p''} m_{cp}m_{cp'}m_{cp''}m_{c'p}m_{c'p'}m_{c'p''} = \sum_{c<c'}\binom{\mathcal{C}_{cc'}}{3};$$
$$N_W(\mathbf{M}) = \sum_{p<p'}\sum_{c<c'<c''} m_{cp}m_{cp'}m_{c'p}m_{c'p'}m_{c''p}m_{c''p'} = \sum_{p<p'}\binom{\mathcal{P}_{pp'}}{3} \qquad \text{(III.4)}$$

respectively. As evident from the definition, countries competitiveness is now measured on a larger number of products.

Since all motifs are defined in terms of products of incidence matrix entries and the latter are treated as independent random variables by our null model, their expectation value can be computed exactly. Thus, we have

$$
\begin{aligned}
\langle N_V\rangle &= \sum_{c=1}^{C}\sum_{c'=c+1}^{C}\sum_{p=1}^{P} p_{cp}p_{c'p},\\
\langle N_\Lambda\rangle &= \sum_{p=1}^{P}\sum_{p'=p+1}^{P}\sum_{c=1}^{C} p_{cp}p_{cp'},\\
\langle N_X\rangle &= \sum_{c=1}^{C}\sum_{c'=c+1}^{C}\sum_{p=1}^{P}\sum_{p'=p+1}^{P} p_{cp}p_{cp'}p_{c'p}p_{c'p'},\\
\langle N_M\rangle &= \sum_{c=1}^{C}\sum_{c'=c+1}^{C}\sum_{p<p'<p''} p_{cp}p_{cp'}p_{cp''}p_{c'p}p_{c'p'}p_{c'p''},\\
\langle N_W\rangle &= \sum_{p=1}^{P}\sum_{p'=p+1}^{P}\sum_{c<c'<c''} p_{cp}p_{cp'}p_{c'p}p_{c'p'}p_{c''p}p_{c''p'}.
\end{aligned} \tag{III.5}
$$

However, when computing the expected value of the generalizations of the V-motifs and Λ-motifs (the Vn and Λn families), higher-order powers of the nodes' degrees appear, since their definition reads $N_{Vn} = \sum_{p=1}^{P}\binom{u_p}{n}$ and $N_{\Lambda n} = \sum_{c=1}^{C}\binom{d_c}{n}$. In these cases, we can exploit the evidence that our degrees can be considered (with a good approximation) gaussian-distributed over the ensemble induced by the BiCM (for example, when considering $N_{V3} = \sum_{p=1}^{P} u_p(u_p-1)(u_p-2)$, the well known result stating that odd central moments of a gaussian distribution are zero can be used to greatly simplify the calculations).

The motifs standard deviation can be calculated by applying the delta method, with $X(\mathbf{M}) = N_m(\mathbf{M})$. While valid in general, this formula assumes a particularly simple form for the Vn and Λn families of motifs. Expliciting it for a few cases will allow us to achieve a double goal: 1) providing a simple expression for the z-scores of the corresponding motifs and 2) showing a limitation of the traditional definition of z-scores. All the calculations will be carried on for the Vn family since they can be easily generalized to the Λn family. Let us start by noticing that Vn motifs are functions of the products degrees exclusively. Now, since in a bipartite network the nodes degrees within each layer are independent random variables, specifying eq. III.2 for the Vn family leads one to write

$$
\sigma_{N_{Vn}} \simeq \sqrt{\sum_{p=1}^{P}\left(\frac{\partial N_{Vn}}{\partial u_p}\right)^2 \sigma_{u_p}^2}, \tag{III.6}
$$

further simplifiable using the binomial result

$$
\frac{\partial\binom{u_p}{n}}{\partial u_p} = \binom{u_p}{n}(H_{u_p} - H_{u_p-n}) \tag{III.7}
$$

with $H_i = \sum_{j=1}^{i}\frac{1}{j}$ being the *i-th harmonic number*. Putting everything together, for $n = 2$ we have

$$
N_V = \sum_{p=1}^{P}\binom{u_p}{2} = \sum_{p=1}^{P}\frac{u_p(u_p-1)}{2}; \tag{III.8}
$$

now, $\langle N_V\rangle = \sum_p\left[\langle u_p^2\rangle - u_p\right]/2$. This allows us to calculate the difference between N_V and $\langle N_V\rangle$ simply as the total number of links variance

$$
N_V - \langle N_V\rangle = -\frac{\sum_{p=1}^{P}\sigma_{u_p}^2}{2} = -\frac{\sigma_L^2}{2} \tag{III.9}
$$

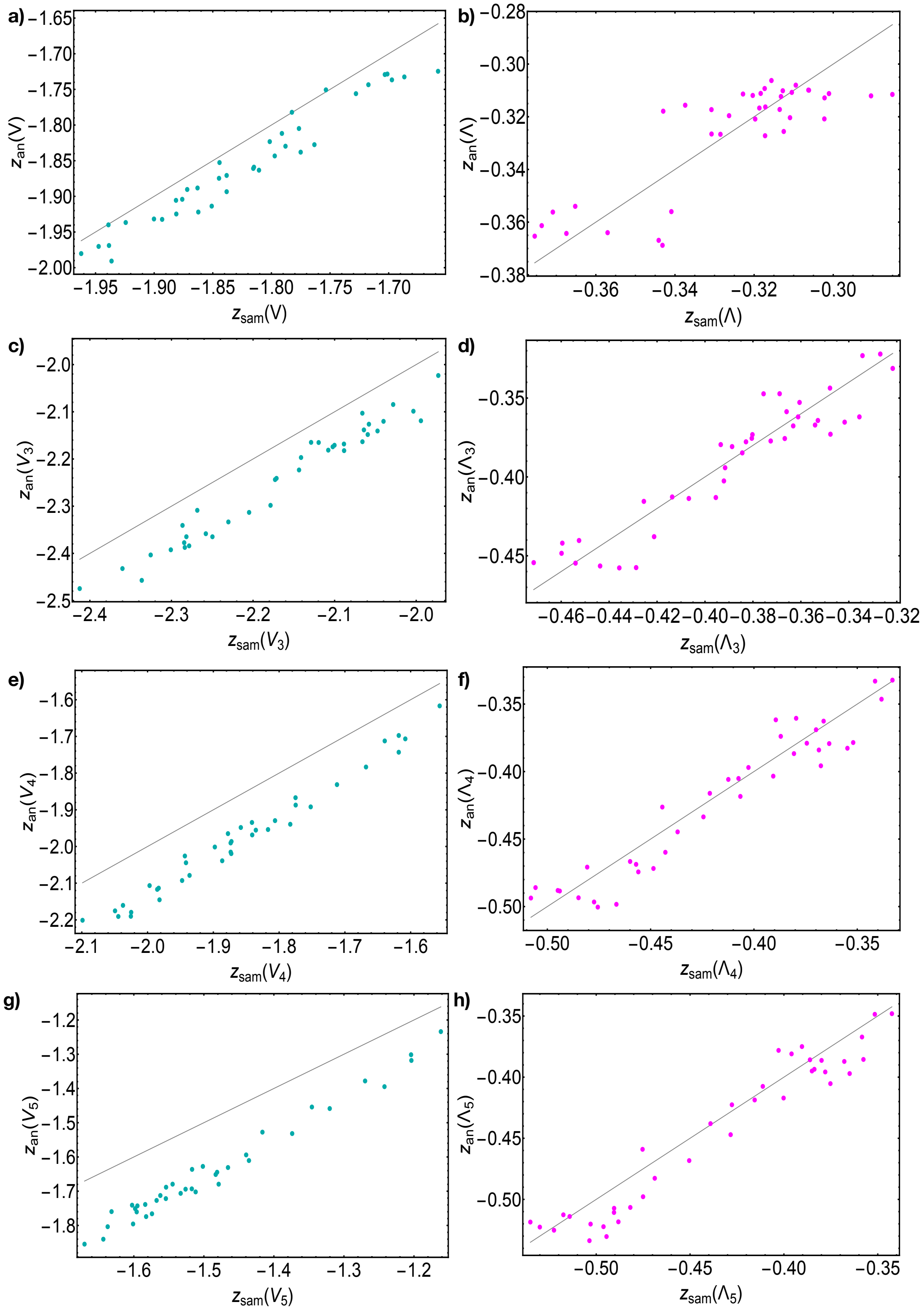

FIG. 2. Comparison between the analytical expressions of the z-scores (on the y-axis) and the corresponding values obtained by explicitly sampling the grandcanonical ensemble induced by the BiCM (on the x-axis), for N_V, N_{V3}, N_{V4}, N_{V5} (●) and N_Λ, $N_{\Lambda 3}$, $N_{\Lambda 4}$, $N_{\Lambda 5}$ (●).

where $\sigma^2_{u_p} = \langle u_p^2\rangle - \langle u_p\rangle^2 = \sum_c \sigma^2_{m_{cp}} = \sum_c p_{cp}(1-p_{cp})$. In order to calculate the standard deviation, we use eq. (III.6) to find

$$\sigma^2_{N_V} \simeq \frac{\sum_{p=1}^P (2u_p-1)^2\sigma^2_{u_p}}{4} \tag{III.10}$$

and finally obtain

$$z_V = \frac{N_V - \langle N_V\rangle}{\sigma_{N_V}} \simeq \frac{-\sigma_L^2}{\sqrt{\sum_{p=1}^P (2u_p-1)^2\sigma^2_{u_p}}}. \tag{III.11}$$

The same procedure can be applied to all the motifs belonging to the Vn and Λn families. More explicitly, in the cases $N_{V3} = \sum_{p=1}^P \binom{u_p}{3}$ and $N_{V4} = \sum_{p=1}^P \binom{u_p}{4}$ the following results hold:

$$z_{V3} \simeq \frac{-\sum_p 3\sigma^2_{u_p}(u_p-1)}{\sqrt{\sum_{p=1}^P (3u_p^2-6u_p+2)^2\sigma^2_{u_p}}} \tag{III.12}$$

and

$$z_{V4} \simeq \frac{-\sum_p [3\sigma^4_{u_p} + \sigma^2_{u_p}(6u_p^2-18u_p+11)]}{\sqrt{\sum_{p=1}^P (4u_p^3-18u_p^2+22u_p-6)^2\sigma^2_{u_p}}}. \tag{III.13}$$

We have also tested the agreement between the analytical expressions of the aforementioned z-scores and the values obtained by explicitly sampling the grandcanonical ensemble induced by the CM. As fig. 2 shows, despite the presence of two approximations, our analytical estimates work quite satisfactorily.

Figs. 3 and 4 show the analysis of the X, M and W-motifs. As for the other motifs previously considered, the three distributions follow a gaussian very closely, whose mean and variance have been calculated on the networks sample (5000 matrices). Again, this can be ascribed to the (generalized) Central Limit Theorem. As a general remark, the three, most complex motifs show higher fluctuations and are less accurately reproduced than the simpler ones (i.e. Vn and Λn).

Beside having provided a simple expression for the z-scores of the Vn and Λn families of motifs, we have also shown that the latter may have a definite sign (negative, in our case): while quite surprising, in cases like this z-scores might still be used to test the agreement between observations and predictions but should be considered *one-sided* statistical tests of significance. This implies that the values enclosing the probabilities of 68%, 95% and 99% no more coincide with $z = \pm 1$, $z = \pm 2$ and $z = \pm 3$, because no more computable on both tails of the reference gaussian distribution. The right z values for one-sided tests are ± 1.65, enclosing a probability of 95%, and ± 2.33, enclosing a probability of 99% [50]. The three more complex motifs (X-motifs, M-motifs and W-motifs) do not have a definite sign.

The reason for the sign definiteness lies in the explicit dependence of the quantities of interest from the chosen constraints, as shown below. Let us consider the Taylor expansion of a quantity of interest $f(x)$ around the expected value $\langle x\rangle$:

$$f(x) = f(\langle x\rangle) + \frac{\partial f}{\partial x}\Big|_{\langle x\rangle}(x-\langle x\rangle) + \frac{\partial^2 f}{\partial x^2}\Big|_{\langle x\rangle}\frac{(x-\langle x\rangle)^2}{2!} + \dots \tag{III.14}$$

After calculating $\langle f\rangle$, the expression can be rewritten as

$$f(\langle x\rangle) - \langle f(x)\rangle = -\frac{\partial^2 f}{\partial x^2}\Big|_{\langle x\rangle}\frac{\sigma_x^2}{2!} - \dots \tag{III.15}$$

Whenever x represents a given, chosen constraint whose expected value on the ensemble is, by definition, equal to the observed one, we obtain

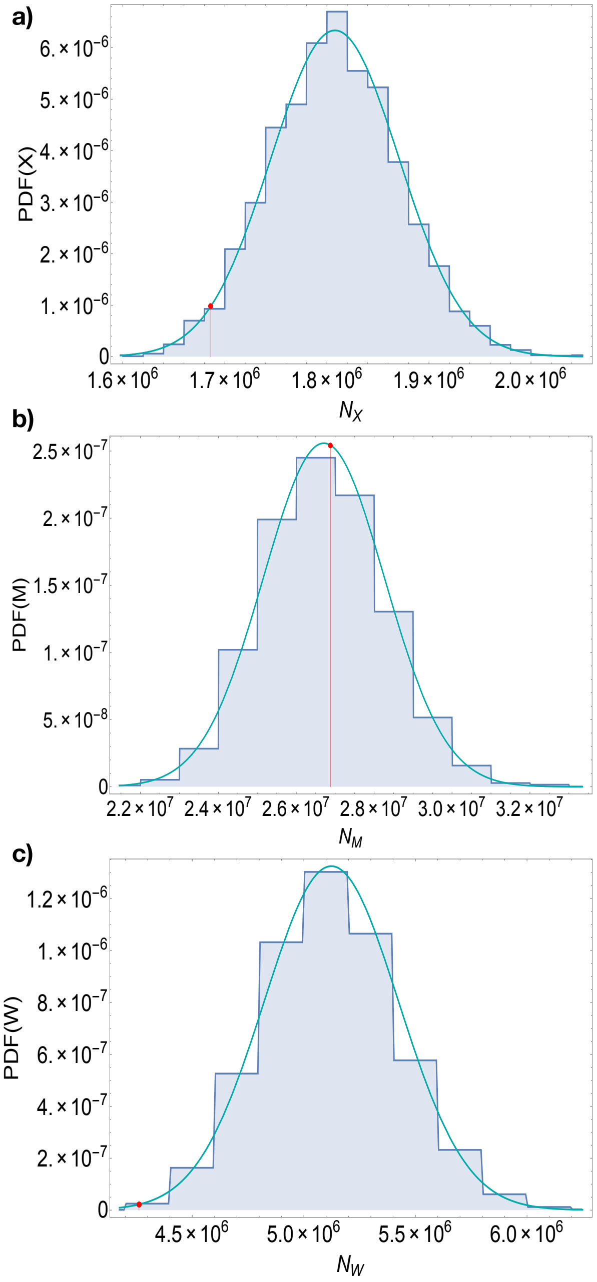

FIG. 3. Analysis of motifs. From top to bottom: ensemble distribution of N_X, N_M and N_W abundance in the year 2000 (fits are obtained by superimposing normal distributions with the sample average and variance; red points represent the observed motifs abundance).

$$f(x) - \langle f(x) \rangle = -\frac{\partial^2 f}{\partial x^2}\Big|_{\langle x \rangle} \frac{\sigma_x^2}{2!} - \dots \tag{III.16}$$

Now, if higher-order moments can be ignored or the function is quadratic in x, the right hand side of the above equation is proportional to the numerator of the f function z-score, whose sign is negative. A simple example is provided by the function x^2, with $x = L$: we obtain $L^2 - \langle L^2 \rangle = \langle L \rangle^2 - \langle L^2 \rangle = -2\frac{\sigma_L^2}{2!} = -\sigma_L^2$, as it should. In the V-motifs case $f(\vec{x}) = f(\{u_p\}) = \sum_p u_p(u_p - 1)/2$ and $N_V - \langle N_V \rangle = -\sum_p \sigma_{u_p}^2/2 = -\sigma_L^2/2$

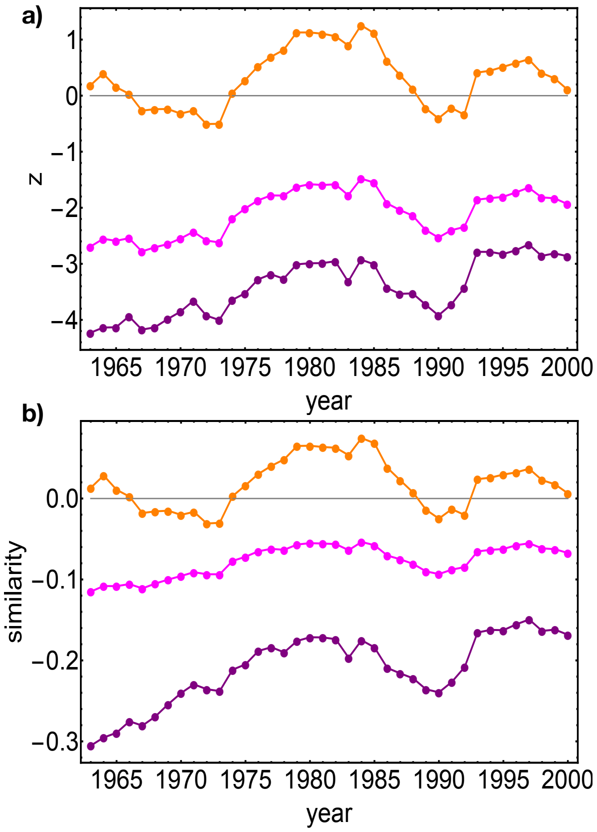

FIG. 4. Analysis of motifs. From top to bottom: z-scores and similarity evolution across our database years of N_M (•), N_X (•), N_W (•).

and analogously for the Λ, V3 and $\Lambda3$ cases (remembering, for the latter, that odd central moments of a gaussian distribution are zero).

This finding has also an obvious interpretation in terms of grandcanonical and microcanonical ensembles. Given a certain set of constraints, the microcanonical approach prescribes them to be exactly satisfied, implying that no statistical fluctuations of the latter can be observed [31]. On the other hand, the grandcanonical approach cannot reduce such fluctuations to zero (as also clearly shown by fig. 1) and the constraints variance will be positive: the dependence of a generic quantity of interest on it, according to the functional form shown before, is reflected in its sign definiteness.

ACKNOWLEDGMENTS

This work was supported by the EU project GROWTHCOM (611272) and the Italian PNR project CRISIS-Lab. The authors thank Giulio Cimini, Matthieu Cristelli and Andrea Tacchella for useful discussions.

AUTHOR CONTRIBUTIONS

FS and RDC analysed the data and prepared all figures. AG wrote the article. TS planned the research and wrote the article. All authors reviewed the manuscript.

ADDITIONAL INFORMATION

The authors declare no competing financial interests.

---

[1] Albert R. & Barabasi. A.-L. Statistical Mechanics of Complex Networks. *Rev. Mod. Phys.* **74**, 47-96 (2002).
[2] Newman M. E. J. The structure and function of complex networks. *SIAM Rev.* **45**, 167 (2003).
[3] Caldarelli G. Scale-free Networks. Complex Webs in Nature and Technology. *Oxford University Press*, Oxford (2007).
[4] Boccaletti S., Latora V., Moreno Y., Chavez M. & Hwang D.-U. Complex Networks: Structure and Dynamics. *Phys. Rep.* **424**(4-5), 175-308 (2006).
[5] Guillaume J.-L. & Latapy M. Bipartite structure of all complex networks. *Inform. Proc. Lett.* **90**(5), 215-221 (2004).
[6] Dormann C. F., Fründ J., Blüthgen N., Gruber B. Indices, Graphs and Null Models: Analyzing Bipartite Ecological Networks. *The Open Ecology Journal* **2**, 7-24 (2009).
[7] Hidalgo C. & Hausmann R. The building blocks of economic complexity *Proc. Nat. Acad. Sci.* **26**(106), 10570-10575 (2009).
[8] Tacchella A., Cristelli M., Caldarelli G., Gabrielli A. & Pietronero L. A New Metrics for Countries' Fitness and Products' Complexity. *Sci. Rep.* **2**, 723, doi:10.1038/srep00723 (2012).
[9] Tacchella A., Cristelli M., Caldarelli G., Gabrielli A. & Pietronero L. Measuring the Intangibles: A Metrics for the Economic Complexity of Countries and Products. *PLoS ONE* **8**(8): e7072. doi:10.1371/journal.pone.0070726 (2013).
[10] Cimini G., Gabrielli A. & Sylos Labini F. The Scientific Competitiveness of Nations. *arXiv:1409.5698* (2014).
[11] Peltomäki M. & Alava M. Correlations in Bipartite Collaboration Networks. *J. Stat. Mech.* **2006**(1), P01010 (2006).
[12] Chung F. & Lu L. Connected Components in Random Graphs with Given Expected Degree Sequences. *Ann. Comb.* **6**, 125-145 (2002).
[13] Caldarelli G., Capocci A., De Los Rios P. & Muñoz M. Scale-Free Networks from Varying Vertex Intrinsic Fitness. *Phys. Rev. Lett.* **89**(25), 258702 (2002).
[14] Park J. & Newman M. E. J. The statistical mechanics of networks. *Phys. Rev. E* **70**, 066117 (2004).
[15] Serrano M. A, Boguna M. & Pastor-Satorras R. Correlations in weighted networks. *Phys. Rev. E* **74**, 055101(R) (2006).
[16] Garlaschelli D. & Loffredo M. I. Maximum likelihood: extracting unbiased information from complex networks. *Phys. Rev. E* **78**, 015101(R) (2008).
[17] Bianconi G. The entropy of network ensembles. *Phys. Rev. E* **79**, 036114 (2009).
[18] Fronczak A. Exponential random graph model. *Encyclopedia of Social Network Analysis and Mining*, Springer-Verlag (2014).
[19] Squartini T. & Garlaschelli D. Analytical maximum-likelihood method to detect patterns in real networks, *New. J. Phys.* **13**, 083001 (2011).
[20] Mastrandrea R., Squartini T., Fagiolo G. & Garlaschelli D. Enhanced reconstruction of weighted networks from strengths and degrees. *New J. Phys.* **16**, 043022 (2014).
[21] Squartini T., Mastrandrea R. & Garlaschelli D. Unbiased sampling of network ensembles. *arXiv:1406.1197* (2014).
[22] Dormann C. F., Gruber B. & Fründ J. Introducing the bipartite Package: Analysing Ecological Networks. *R News* **8**(2), 8-11 (2008).
[23] Strona G., Nappo D., Boccacci F., Fattorini S. & San-Miguel-Ayanz J. A fast and unbiased procedure to randomize ecological binary matrices with fixed row and column totals. *Nat. Comm.* **5**(4114), doi:10.1038/ncomms5114 (2014).
[24] Kitsak M. & Krioukov D. Hidden Variables in Bipartite Networks. *Phys. Rev. E* **82**, 026114 (2011).
[25] Dormann C. F., Strauss R. A method for detectingmodules in quantitative bipartite networks. *Methods in Ecology and Evolution* **5**, 90-98 (2014).
[26] Squartini T., Fagiolo G. & Garlaschelli D. Randomizing world trade. I. A binary network analysis. *Phys. Rev. E* **84**, 046117 (2011).
[27] Squartini T., Fagiolo G. & Garlaschelli D. Randomizing world trade. II. A weighted network analysis. *Phys. Rev. E* **84**, 046118 (2011).
[28] Squartini T. & Garlaschelli D. Triadic Motifs and Dyadic Self-Organization in the World Trade Network. *Lec. Notes Comp. Sci.* **7166**, 24-35 (2012).
[29] Musmeci N., Battiston S., Puliga M. & Gabrielli A. Bootstrapping topology and systemic risk of complex network using the fitness model. *J. Stat. Phys.* **151**, 720-734 (2013).
[30] Caldarelli G., Chessa A., Pammolli F., Gabrielli A. & Puliga M. Reconstructing a credit network. *Nat. Phys.* **9**, 125-126, doi:10.1038/nphys2580 (2013).

[31] Tumminello M., Micciché S., Lillo F., Piilo J., Mantegna R.N. Statistically Validated Networks in Bipartite Complex Systems. *PLoS ONE* **6**(3): e17994. doi:10.1371/journal.pone.0017994 (2011).

[32] Fronczak A. & Fronczak P. Statistical mechanics of the international trade network. *Phys. Rev. E* **85**, 056113 (2012).

[33] Serrano M. A. & Boguna M. Topology of the world trade web. *Phys. Rev. E* **68**, 015101(R) (2003).

[34] Fagiolo G., Reyes J. & Schiavo S. The evolution of the world trade web: a weighted-network analysis. *Journal of Evolutionary Economics* **20**(4), 479-514 (2010).

[35] Barigozzi M., Fagiolo G. & Garlaschelli D. Multinetwork of international trade: A commodity-specific analysis. *Phys. Rev. E* **81**, 046104 (2010).

[36] Mastrandrea R., Squartini T., Fagiolo G. & Garlaschelli D. Intensive and extensive biases in economic networks: reconstructing the world trade multiplex. *arXiv:1402.4171* (2014).

[37] http://www.nber.org/data/

[38] Feenstra R. C., Lipsey R. E., Deng H., Ma A. C. & Mo H. World Trade Flows: 1962-2000. *National Bureau of Economic Research working paper 11040*, doi:10.3386/w11040 (2005).

[39] Milo R., Shen-Orr S., Itzkovitz S., Kashtan N., Chklovskii D. & Alon U. Network motifs: simple building blocks of complex networks. *Science* **298**, 824-827 (2002).

[40] Newman M. E. J. Assortative mixing in networks. *Phys. Rev. Lett.* **89**, 208701 (2002).

[41] Almeida-Neto M., Guimaraes P., Guimaraes P. R. Jr., Loyola R. D. & Ulrich W. A consistent metric for nestedness analysis in ecological systems: reconciling concept and measurement. *Oikos* **8**(117), 1227-1239 (2008).

[42] Bastolla U., Fortuna M. A., Pascual-Garcia A., Ferrera A., Luque B. & Bascompte J. The architecture of mutualistic networks minimizes competition and increases biodiversity. *Nature* **7241**(458), 1018-1020 (2009).

[43] Staniczenko P. P. A., Kopp J. & Allesina S. The ghost of nestedness in ecological networks. *Nature Communications* **4**(139), doi: 10.1038/ncomms2422 (2013).

[44] Jonhson S., Dominguez-Garcia V. & Munoz M. A. Factors Determining Nestedness in Complex Networks. *PLoS ONE* **8**(9), doi:10.1371/journal.pone.0074025 (2013).

[45] Pugliese E., Zaccaria A. & Pietronero L. On the convergence of the Fitness-Complexity Algorithm. *arXiv:1410.0249* (2014).

[46] Milo R., Itzkovitz S., Kashtan N., Levitt R., Shen-Orr S., Ayzenshtat I., Sheffer M. & Alon U. Superfamilies of evolved and designed networks. *Science* **303**, 1538-1542 (2004).

[47] Caldarelli G., Cristelli M., Gabrielli A., Pietronero L., Scala A. & Tacchella A. A Network Analysis of Countries' Export Flows: Firm Grounds for the Building Blocks of the Economy. *PLoS ONE* **7**(10): e47278. doi:10.1371/journal.pone.0047278 (2012).

[48] Squartini T. & Garlaschelli D. Stationarity, non-stationarity and early warning signals in economic networks. *Journal of Complex Networks*, doi: 10.1093/comnet/cnu012 (2014).

[49] Mastrandrea R., Squartini T., Fagiolo G., Garlaschelli D. Reconstructing the world trade multiplex: the role of intensive and extensive biases. *Phys. Rev. E* **90**, 062804 (2014).

[50] Freund J. E. Modern Elementary Statistics. *Prentice hall* (1984).
